# Polyphenols of Perilla frutescens of the family Lamiaceae identified by tandem mass spectrometry

**DOI:** 10.18699/VJGB-22-78

**Published:** 2022-11

**Authors:** M.P. Razgonova, N.G. Kon’kova, A.M. Zakharenko, K.S. Golokhvast

**Affiliations:** Federal Research Center the N.I. Vavilov All-Russian Institute of Plant Genetic Resources (VIR), St. Petersburg, Russia Far Eastern Federal University, Vladivostok, Russia; Federal Research Center the N.I. Vavilov All-Russian Institute of Plant Genetic Resources (VIR), St. Petersburg, Russia; Siberian Federal Scientific Centre of Agro-BioTechnology of the Russian Academy of Sciences, Krasnoobsk, Novosibirsk Region, Russia Tomsk State University, Tomsk, Russia; Federal Research Center the N.I. Vavilov All-Russian Institute of Plant Genetic Resources (VIR), St. Petersburg, Russia Far Eastern Federal University, Vladivostok, Russia

**Keywords:** Perilla frutescens, HPLC–MS/MS, tandem mass spectrometry, phenolic compounds, triterpene acids, lignans, Perilla frutescens, ВЭЖХ–MС/MС, тандемная масс-спектрометрия, фенольные соединения, тритерпеновые кислоты, лигнаны

## Abstract

Perilla frutescens is mainly cultivated as an oilseed crop. Perilla seeds contain 40–53 % of oil, 28 % of protein. The growing season is 100–150 days. In Russia, perilla is grown in the Far East, where the yield is 0.8–1.2 t/ha. Perilla of different geographical origin has its own special, sharply different features that characterize two geographical groups: Japanese and Korean-Chinese. These groups differ from each other in the length of the growing season, the height of plants, the color of the stem, the surface and the size of the leaves, the shape of the bush, the shape and size of the inflorescences, the size of the cups, the size and color of the seeds. P. frutescens contains a large number of polyphenolic compounds that are biologically active components. The purpose of this research was a metabolomic study of extracts from leaves of P. frutescens obtained from the collection of Federal Research Center the N.I. Vavilov All-Russian Institute of Plant Genetic Resources, grown on the fields of the Far East Experiment Station – Branch of Federal Research Center (Primorsky Krai, Russia). To identify target analytes in extracts, HPLC was used in combination with an ion trap. Preliminary results showed the presence of 23 biologically active compounds corresponding to P. frutescens. In addition to the reported metabolites, a number of metabolites were newly annotated in P. frutescens. There were hydroxycoumarin Umbelliferone; triterpene Squalene; omega-3 fatty acid Stearidonic [Moroctic] acid; higher-molecular-weight carboxylic acid: Tetracosenoic acid and Salvianic acid C; lignan Syringaresinol and cyclobutane lignan Sagerinic acid, etc. A wide range of biologically active compounds opens up rich opportunities for the creation of new drugs and dietary supplements based on extracts of perilla of the family Lamiaceae, subfamily Lamioideae, tribe Satureji and subtribe Perillinae.

## Abstract (ru)

Perilla frutescens получила применение в основном как масличная культура. Семена периллы содержат 40–53 % масла, 28 % белка. Вегетационный период составляет 100–150 дней. В России периллу выращивают на Дальнем Востоке, где урожайность достигает 0.8–1.2 т/га. Это растение короткого дня, поэтому большинство форм не цветет в условиях средней полосы России. Перилла различного географического происхождения имеет свои особенные, резко отличные признаки, характеризующие две географические группы: японскую и корейско-китайскую. Эти группы различаются длиной вегетационного периода, а также по высоте растений, окраске стебля, поверхности и величине листьев, форме куста, форме и размеру соцветий, величине чашечек и цвету семян. Perilla frutescens содержит большое количество полифенольных соединений, которые являются биологически активными компонентами. Цель данной работы состояла в метаболомном исследовании экстрактов из листьев P. frutescens, полученных из коллекции Всероссийского института генетических ресурсов растений им. Н.И. Вавилова и выращенных на полях его Дальневосточной опытной станции (Приморский край, Россия). Для идентификации целевых аналитов в экстрактах использовали метод высокоэффективной жидкостной хроматографии в сочетании с ионной ловушкой. Предварительные результаты показали наличие 23 биологически активных соединений, соответствующих виду P. frutescens. В дополнение к упомянутым метаболитам, в экстрактах P. frutescens был впервые обнаружен ряд соединений. Это кумарин умбеллиферон; тритерпен сквален; стеаридо- новая кислота; высокомолекулярные карбоновые кислоты: тетракозановая кислота и сальвиановая кислота C; лигнан сирингарезинол; циклобутановый лигнан сагериновая кислота и др. Широкий спектр биологически ак- тивных соединений открывает богатые возможности для создания новых лекарственных средств и биологически активных добавок на основе экстрактов периллы семейства Lamiaceae, подсемейства Lamioideae, трибы Satureji и подтрибы Perillinae.

## Introduction

This research presents a detailed study of the metabolomic composition of Perilla frutescens leaves. Perilla frutescens L. is an annual plant belonging to the mint family Lamiaceae, subfamily Lamioideae, tribe Satureji and subtribe Perillinae (Zhou et al., 2014). Perilla is widely cultivated in Asian countries such as China, Japan, South Korea and India for its oils and leaves used in cooking. Perilla has also been cultivated in Russia in the Far East since the 1930s to obtain high quality oil.

Perilla is a heat-loving and moisture-loving plant. It requires fertile soils. Perilla is a short-day plant, so most forms do not bloom in the conditions of Central Russia or bloom only in late autumn. Perilla of different geographical origin has its own special, sharply different features that characterize two geographical groups: Japanese and Korean-Chinese. These groups differ from each other in the length of the growing season, the height of plants, the color of the stem, the surface and the size of the leaves, the shape of the bush, the shape and size of the inflorescences, the size of the cups, the size and color of the seeds. Perilla leaves are commonly used for their antioxidant, anti-allergic, antimicrobial, anti-tumor and anti-cancer effects due to the presence of phenolic compounds including rosemary acid, essential oil and vitamins (Ahmed, 2019).

The fatty acid composition of perilla oil is characterized by the presence of five main fatty acids. On average, perilla oil contains (% of the total fatty acids): palmitic acid – 5.9, stearic acid – 1.8, oleic acid – 15.3, linoleic acid – 12.4, α-linolenic acid – 61.9. The increased content of polyunsaturated fatty acids – up to 90 % – indicates a high biological activity of perilla oil. By their properties, these acids are close to vitamins (vitamin F), which are not synthesized in the human organism. In terms of the sum of these acids, perilla oil even exceeds many varieties of flax and hemp. It is important to observe the ratio of ω-3 and ω-6 fatty acids in the diet. The optimal ratio of ω-3 and ω-6 fatty acids is 1:4 (Banno et al., 2004; Gu et al., 2009; Meng et al., 2009). Since unsaturated fatty acids and α-linolenic acid are thought to have various beneficial effects on the human health, such as lowering serum cholesterol and triglyceride levels, reducing the risk of colon cancer, and preventing overgrowth of visceral adipose tissue (Longvah et al., 2000), perilla seed oil is considered to be of high quality.

Many bioactive compounds from various chemical groups have been identified from the leaves and seeds of extract of P. frutescens. P. frutescens is used as a spice as well as in medicine and consists of several chemotypes that refer to the essential oils chemical composition. A chemotype containing perillaldehyde is a major component of the essential oil that is most effective as a sedative in China’s traditional medicine. Honda et al. (1986) fractionated MeOH extract of P. frutescens to presence of stigmasterol and perillaldehyde. Also, several studies showed the presence of other flavonoids such as apigenin and luteolin, and phenolic compounds such as caffeic acid and rosmarinic acid (Lee et al., 2013; Kauffmann et al., 2016).

Thus, we isolated and investigated the structure of phenolic compounds and triterpenic acids from P. frutescens leaves. A total of 23 biologically active compounds: 13 phenolic compounds, omega-3-fatty acids, lignans, sterols and triterpenic acids were identified using tandem ion trap mass spectrometry.

## Materials and methods

Perilla frutescens leaves served as the object of the study. The variety ‘Novinka’ from the collection of Federal Research Center the N.I. Vavilov All-Russian Institute of Plant Genetic Resources (VIR) was grown on the fields of the Far East Experiment Station – Branch of VIR, Primorsky Territory (N 43°21′34″, E 132°11′19″; yellow-brown soil). This is the only perilla oilseed variety listed in the State Register of the Russian Federation. The variety ‘Novinka’ is a mediumripened variety of the Korean-Chinese ecological group with a growing season length of 106 days and an oil content of 49 %, the yield is 0.8–1.2 t/ha.

The leaves were harvested at the end of August, 2020. Weather conditions were favorable for the perilla growth and development. The average air temperature in August was 20 °C, the amount of precipitation was 250 mm. All samples morphologically corresponded to the pharmacopoeial standards of the Pharmacopoeia of the Eurasian Economic Union (2020).

Chemicals and reagents. HPLC-grade acetonitrile was pur- chased from Fisher Scientific (Southborough, UK), MS-grade formic acid was obtained from Sigma-Aldrich (Steinheim, Germany). Ultra-pure water was prepared from a SIEMENS ULTRA clear (SIEMENS water technologies, Germany), and all other chemicals were analytical grade.

Fractional maceration. To obtain highly concentrated extracts, fractional maceration was applied. In this case, the total amount of the extractant (ethyl alcohol of reagent grade) was divided into 3 parts and was consistently infused on perilla with the first part, then with the second and third, correspondingly. The infusion time of each part of the extractant was 7 days. Fractional maceration technique was applied to obtain highly concentrated extracts (Azmir at al., 2013). From 300 g of the fresh sample, 50 g of leaves of P. frutescens were selected for maceration. The total amount of the extractant (ethyl alcohol of reagent grade) was divided into three parts and consistently infused to the leaves with the first, second and third parts. The solid–solvent ratio was 1:20. The infusion of each part of the extractant lasted 7 days at room temperature.

Liquid chromatography. HPLC was performed using Shimadzu LC-20 Prominence HPLC (Shimadzu, Japan)equipped with a UV-sensor and a Shodex ODP-40 4E reverse phase column for multicomponent mixtures separation. The gradient elution program was as follows: 0.01–4 min, 100 % C2H3N; 4–60 min, 100–25 % C2H3N; 60–75 min, 25–0 % C2H3N; control washing 75–120 min 0 % C2H3N. The entire HPLC analysis was done with a UV detector at wavelengths of 230 and 330 nm; the temperature corresponded to 17 °C. The injection volume was 1 ml.

Mass spectrometry. MS analysis was performed on an ion trap amaZon SL (BRUKER DALTONIKS, Germany) equipped with an ESI source in negative and positive ion mode. The optimized parameters were obtained as follows: ionization source temperature: 70 °C, gas flow: 4 l/min, nebulizer gas (atomizer): 7.3 psi, capillary voltage: 4500 V, end plate bend voltage: 1500 V, fragmentary: 280 V, collision energy: 60 eV. An ion trap was used in the scan range m/z 100–1.700 for MS and MS/MS.

Data collection was controlled by Windows software for BRUKER DALTONIKS. All experiments were repeated three times. A four-stage ion separation mode (MS/MS mode) was implemented.

## Results and discussion

**Fig. 1.**
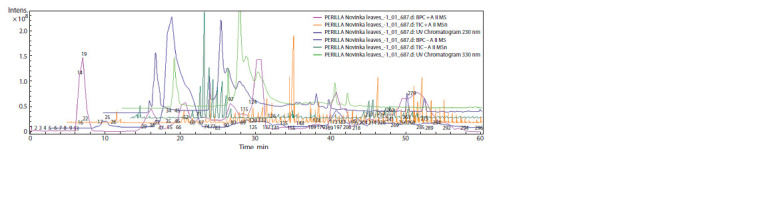
Chemical profiles of the P. frutescens sample (Primorsky Territory, Russia) represented in a total ion chromatogram from EtOH-extract

Five of the most EtOH extracts of P. frutescens were selected. All of them had a rich polyphenolic and triterpene composition. High accuracy mass spectrometric data were recorded on an ion trap amaZon SL BRUKER DALTONIKS equipped with an ESI source in the mode of negative/positive ions. The four-stage ion separation mode (MS/MS mode) was implemented. All the chemical profiles of the samples were obtained by the HPLC – ESI – MS/MS method. A total of 300 peaks were detected in the chromatogram (Fig. 1).

The combination of both ionization modes (positive and negative) in MS full scan mode is giving certainty to the molecular mass determination. The negative ion mode provides the highest sensitivity and results in limited fragmentation making it most suited to infer the molecular mass of the separated polyphenols especially in cases where concentration is low. A tentative identification of compounds was carried out using comparisons of the m/z values, the RT and the fragmentation patterns with the MS2 spectral data taken from the literature (Banno et al., 2004; Vallverdu-Queralt et al., 2012; Zhou et al., 2014; Spinola et al., 2015; Cirlini et al., 2016; Pandey et al., 2016; Sharma et al., 2016; Marzouk et al., 2018; Sun L. et al., 2019; Goufo et al., 2020; etc.) or the data bases (MS2T, MassBank, HMDB). A unifying system table of the molecular masses of the target analytes isolated from the EtOH-extract of P. frutescens was compiled for ease of identification (see the Table). The 23 compounds are shown in the Table. Some of them belong to different polyphenolic families: anthocyanidins, flavones, hydroxycinnamic acids, hydroxybenzoic acids, lignans.

**Table 1.**
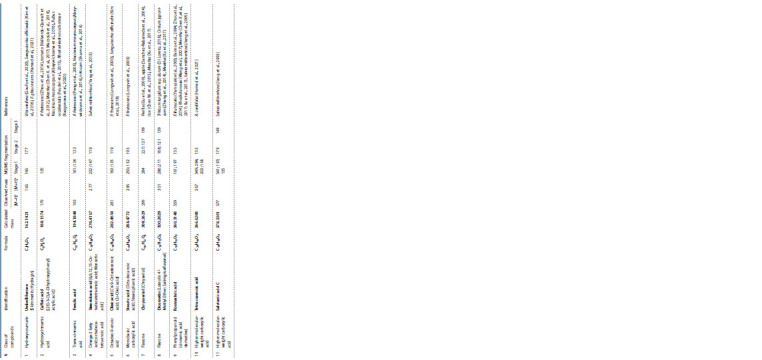
Biologically active substances identified from the EtOH-extracts of P. frutescens

**Table 1end.**
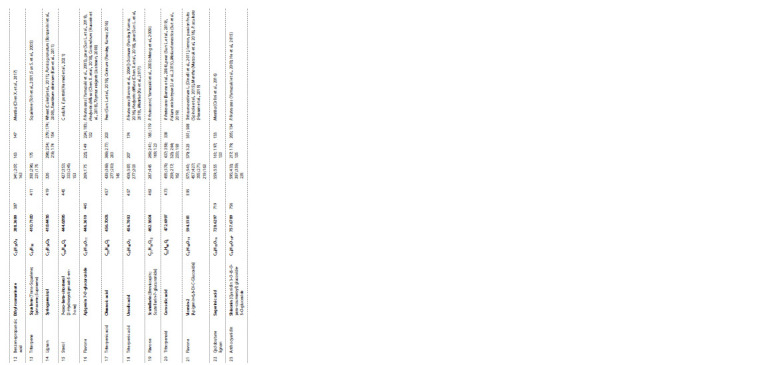
Biologically active substances identified from the EtOH-extracts of P. frutescens

In addition to the reported metabolites, a number of metabolites were newly annotated in P. frutescens. The newly annotated metabolites were hydroxycoumarin Umbelliferone; triterpene Squalene; omega-3 fatty acid: Stearidonic [Moroctic] acid; higher-molecular-weight carboxylic acids: Tetracosenoic acid and Salvianic acid C; cyclobutane lignan Sagerinic acid; sterol 7-oxo-beta-sitosterol [3-Hydroxystigmast-5-en-7-one]; flavone Vicenin-2 [Apigenin-6,8-Di-C-Glucoside].

A total of 13 polyphenol compounds have been identified (see the Table). The flavones Chrysoeriol, Diosmetin, Apigenin 7-O-glucuronide, Scutellarin, Vicenin-2 have already been characterized as a component of P. frutescens. This identification was satisfactory according to the studied references in P. frutescens (Yamazaki et al., 2003; Gu et al., 2009; Meng et al., 2009; Zhou et al., 2014), Triticum aestivum L. (Di Loreto et al., 2018), apple (Sanchez-Rabaneda et al., 2004), rice (Chen W. et al., 2013), Mentha (Xu et al., 2017), Cirsium japonicum (Zhang et al., 2014), etc.

**Fig. 2.**
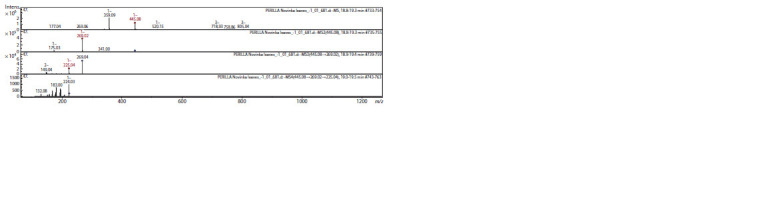
CID-spectrum of Apigenin-7-O-glucuronide from extracts of P. frutescens, m/z 445.08.

The CID-spectrum (collision induced dissociation spectrum) in negative ion modes of Apigenin-7-O-glucuronide from extracts of P. frutescens is shown in Figure 2. The [M–H]– ion produced three fragment ions at m/z 269.02, m/z 341.00, m/z 175.03 (see Fig. 2). The fragment ion with m/z 269.02 yields two daughter ions at m/z 225.04, and m/z 149.04. The fragment ion with m/z 225.04 yields three daughter ions at m/z 224.03, m/z 183.00, and m/z 132.08. It was identified in the bibliography in extracts from P. frutescens (Yamazaki et al., 2003), pear (Sun L. et al., 2019), Hedyotis diffusa (Chen X. et al., 2018), Coriandrum (Hussein et al., 2018), Thymus vulgaris (Justesen, 2000).

**Fig. 3.**
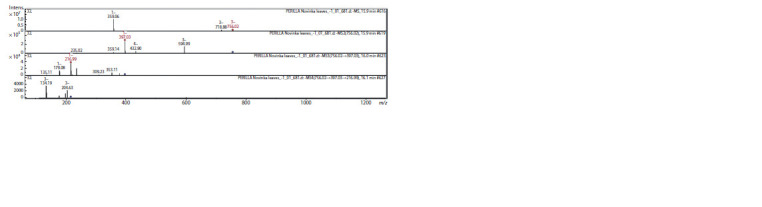
CID-spectrum of [Cyanidin 3-O-(6-O-para-coumaroyl) glucoside-5-O-glucoside] from extracts of P. frutescens, m/z 756.02.

The anthocyanin Shisonin [Cyanidin 3-O-(6-O-para-coumaroyl) glucoside-5-O-glucoside] was found in extracts of P. frutescens (Fig. 3). The Shisonin CID-spectrum in negative ion mode is shown in Figure 3.

The [M–H]– ion produced five fragment ions at m/z 397.03, m/z 432.90, m/z 594.99, m/z 359.14, and m/z 235.02 (see Fig. 3). The fragment ion with m/z 397.03 yields five daughter ions at m/z 216.99, m/z 309.23, m/z 353.11, m/z 179.08, and m/z 135.11. The fragment ion with m/z 216.99 yields two daughter ions at m/z 204.63 and m/z 134.19. These results were in agreement with bibliography of P. frutescens (Yamazaki et al., 2003; He et al., 2015).

## Conclusion

The extracts of P. frutescens from the N.I. Vavilov All-Russian Institute of Plant Genetic Resources contain a large number of polyphenolic complexes, which are biologically active compounds. For the most complete and safe extraction, the method of maceration with EtOH was used. To identify target analytes in extracts, tandem mass spectrometry, HPLC and the ion trap were used. The preliminary results showed the presence of 23 bioactive compounds corresponding to P. frutescens. In addition to the reported metabolites, a number of metabolites were newly annotated in P. frutescens leaves. There were hydroxycoumarin Umbelliferone; triterpene Squalene; omega-3 fatty acid Stearidonic [Moroctic] acid; higher-molecularweight carboxylic acids: Tetracosenoic acid and Salvianic acid C; lignan Syringaresinol and cyclobutane lignan Sagerinic acid; sterol 7-oxo-beta-sitosterol [3-Hydroxystigmast-5-en- 7-one]; benzenepropanoic acid Ethyl rosmarinate; flavones Diosmetin and Vicenin-2 [Apigenin-6,8-Di-C-Glucoside].

The findings may support future research into the production of various pharmaceutical and dietary supplements containing P. frutescens extracts. A wide variety of bioactive compounds opens up rich opportunities for the creation of new drugs and bioactive additives based on extracts from mint family Lamiaceae, subfamily Lamioideae, tribe Satureji and subtribe Perillinae. In continuation of the study, we are planning to determine the quantitative content of the identified substances.

## Conflict of interest

The authors declare no conflict of interest.
